# Sleep/wake movement velocities, trajectories and micro-arousals during maturation in rats

**DOI:** 10.1186/s12868-017-0343-6

**Published:** 2017-02-07

**Authors:** Gideon Gradwohl, Nadja Olini, Reto Huber

**Affiliations:** 1 Child Development Center, University Children’s Hospital Zurich, Zurich, Switzerland; 2 Neuroscience Center Zurich (ZNZ), University and ETH Zurich, Zurich, Switzerland; 3 Departement of Computer Sciences, Lev Academic Center, Jerusalem, Israel; 4 Department of Child and Adolescent Psychiatry and Psychotherapy, Psychiatric Hospital, University of Zürich, Zurich, Switzerland

**Keywords:** State dynamic, EEG, Maturation, Lobes, Rat

## Abstract

**Background:**

Sleep is regulated by two main processes. The circadian process provides a 24-h rhythm and the homeostatic process reflects sleep pressure, which increases in the course of wakefulness and decreases during sleep. Both of these processes undergo major changes during development. For example, sleep homeostasis, measured by means of electroencephalogram (EEG) slow-wave activity (SWA, EEG power between 0.5 and 4.5 Hz), peaks around puberty and decreases during adolescence. In humans and rats these changes have been related to cortical maturation. We aimed to explore whether additional parameters as state dynamic (dynamic of sleep/wake behavior) parameters of movement velocity, trajectories and micro-arousals provide markers of rat maturation. The state dynamics reflect the stability of sleep within a specific sleep stage. We applied a state space technique (SST), a quantitative and unbiased method, based on frequency band ratios of the EEG to analyze the development of different sleep/wake states and state dynamics between vigilance states. EEG of recording electrodes at the frontal and parietal lobe were analyzed using conventional scoring criteria and SST.

**Results:**

We found that movement velocity, trajectories between sleep states and micro-arousals changed as an inverse U-shaped curve across maturation. At all ages, movement velocity over the frontal lobe is higher compared to the parietal lobe, while the number of trajectories and micro-arousals are reduced. Furthermore, we showed that SWA correlates negatively with movement velocity and the number of micro-arousals. The velocity in the parietal lobe correlates positively with the number of micro-arousals. As for SWA, trajectories seem primarily to depend on sleep homeostasis regulatory mechanisms while the movement velocity seems to be modulated by other sleep regulators like the circadian rhythms.

**Conclusions:**

New insights in sleep/wake state dynamics are established with the SST, because trajectories, micro-arousals and velocities are not evident by traditional scoring methods. These dynamic measures may represent new indicators for changes in sleep regulatory processes across maturation.

**Electronic supplementary material:**

The online version of this article (doi:10.1186/s12868-017-0343-6) contains supplementary material, which is available to authorized users.

## Background

Slow wave activity (SWA), defined as the electroencephalographic (EEG) power between 0.5 and 4.5 Hz in non-rapid eye movement (NREM) sleep, is a well-known signature of sleep pressure [1] and reflects the homeostatic regulation of sleep [1]. The level of SWA at sleep onset is a function of the duration of prior wakefulness and SWA decreases in the course of sleep. In addition to the homeostatic component of sleep regulation, the sleep/wake cycle is controlled by circadian rhythms in humans [1] and in rodents [2]. The major circadian pacemaker is located in the suprachiasmatic nuclei (SCN) of the anterior hypothalamus, which includes neurons driven by a transcriptional-translational loop that fire in a 24 h cycle [3].

On the neuronal level slow waves are reflected by a synchronous slow oscillation (<1 Hz) of the activity of large groups of cortical neurons. More specifically, during a slow oscillation neuronal activity changes between two distinct states, each lasting a few hundreds of milliseconds: The up states are reflected by neuronal firing, which ceases during the down states [4]. SWA seems to reflect network connectivity since increased connectivity facilitates synchronization. Vyazovskiy et al. [5] have shown that under high sleep pressure, when SWA is increased, network synchronization is high, thus up- and down states occur very synchronously across large populations of neurons, giving rise to large amplitude slow waves. The same study shows that when sleep pressure has dissipated, the reduction in SWA goes along with a reduction in network synchronization. Interestingly, electrophysiological (electrically evoked responses) and molecular markers (e.g. GluR1-containing AMPA receptor) of synaptic strength in the rat follow a similar time course: they are increased after periods of wakefulness and decrease in the course of sleep [6].

SWA during maturation has been shown to follow an inverted U-shaped curve in humans, which was recently replicated in rodents. SWA decreases during adolescence in humans [7–9] mice [10], and rats [9, 11–13], and it was hypothesized that this phenomena is due to elimination of cortical synapses [8]. This reduction in connectivity might result in a reduction of network synchronization and as a consequence in a reduction of SWA. However, according to other studies [14, 15], this decline in SWA is not due to a net pruning of synapses and other methods of cortical refinement should be investigated. For example, recently it was shown [16], based on a large computer model of thalamic-cortical neurons, that a refinement of the synaptic connections was sufficient to explain a decrease of SWA. In the pre-refinement state, the synaptic connections were homogenous, but in the post-refinement state, neurons connected to neurons at preferred orientation with similar receptive fields. This modification alone was enough to mimic the experimental results.

In the cortex, under some conditions, the EEG may be heterogeneous because the brain produces a temporal uncoupling of electrical activity between different cortical regions, indicating that sleep is not only a global phenomenon but also a local brain process [15, 17]. In dolphins, for example, verified by EEG recording, one hemisphere can be awake while the other is asleep [18]. In humans, separated states of sleep-awake are present during “sleep walking”, when some cortical region are awake while others are asleep [19]. Moreover, regional differences in SWA have also been presented in mice [20].

NREM sleep is sometimes shortly interrupted by a phenomenon called micro-arousals. The American Sleep Disorders Association (ASDA) has declared the criteria for micro-arousals in sleep [21] as rapid modification in EEG frequency during NREM, which can include theta and alpha activity, and/or frequencies higher than 16 Hz but not spindles. It can be accompanied by an increase in electromyographic (EMG) activity, a temporary rise in heart rate or body movements. A micro-arousal must be preceded by uninterrupted sleep. In rapid eye movement (REM) sleep, the criteria for micro-arousals were a temporary disappearance of eye movements and presence of alpha activity. Micro-arousals are often involved in the pathophysiology of sleep disorders [22–24], indicating that they are a harmful feature for sleep. The functional importance of micro-arousal in sleep, and particularly in NREM sleep, is to confirm the reversibility of sleep [25, 26] and to connect the sleeper to outer world danger. Otherwise, the sleeping organism would stay in a coma.

The traditional EEG scoring method simply identifies discrete vigilance states in a time window (4–10 secs) and often exclude or dilute events through averaging. However, cortical activity and behavior can change quite rapidly and it is necessary to analyze sleep/wake as continuum as a function of time. Therefore, the 2-dimensional (2D) state space technique (SST) was introduces as a state dynamic method. SST provides an unbiased approach for the use as a non-categorical method of comparison and quantitative analysis of sleep/wake states [27–32]. While SWA is calculated using one frequency band, SST employs the ratio of two frequency bands, yielding a normalized value.

We hypothesize that SST can highlight the richness of sleep state transitions and the stability of the states, allowing insights into sleep intensity and stability. It should be appropriate to explore state dynamic of rodents with high temporal resolution of 1 s; the movement’s velocity of transitions between different vigilance states, the trajectories and the micro-arousals short time events. We assume that SWA is not appropriate to evaluate state dynamics since its calculation rely on longer time windows (e.g. 4 s) than SST, not suitable to catch fast variations of the EEG signal. The movement velocity describes how fast the EEG signal is altering its frequency, which characterizes, among other physiological parameters, the vigilance states. Trajectories and micro-arousals indicate the number of vigilance states changes in a certain time period. Micro-arousals define the number of short alteration between vigilance states and trajectories up to two times longer ones. These state dynamics parameters are essential for state stability evaluation which go beyond the classical vigilance state (e.g. in 4 s epochs).They provide information about brain states on shorter time scale. Specifically, a low number of trajectories and micro-arousals describes a high stability of a vigilance state. On the other hand, fast movement velocity can return the EEG to its initial state even if the number of trajectories and micro-arousals is high.

The current study uses SST to analyze the impact of maturity on sleep/wake dynamics and sleep stage stability. It extends a previous study [27] in which sleep intensity and quality were presented, based on one recording electrode, in rats at age P34 and P71. In the present study, state dynamic parameters of young rats (P26, P30, P35 and P38) were measured during a very early period of brain development. These state dynamic parameters were analyzed for EEGs at two distinct cortical regions since topographical differences can be expected. According to our knowledge, state dynamics analysis was never applied at rats with so young ages.

## Methods

We used the same rats as Olini et al. [11] and the following two paragraphs describe the methods shortly.

### Surgical procedures

Sprague–Dawley male rats at an age of 22 days were delivered and placed immediately in the recording box, 3–5 days before the begin of recording. The animals were epidurally implanted with gold-plated miniature screws under isoflurane anesthesia for EEG recordings. In addition, EMG recordings, two gold wires were inserted bilaterally into the neck muscles, connected to steel wires and fixed to the skull. After the surgery, the rats remained with their normal weight and no cranial damage of the brain was distinguished.

### Sleep deprivation (SD)

In order to perform sleep deprivation, novel objects were introduced into the cages of the rats to engage them in exploratory behavior. Also the cage change for cleaning purposes was performed during this time. The rats were never touched except at the time of cage change [20].

### Electrocortical recordings

During 20 consecutive days of data recording the animals stayed connected by a fine cable to a swivel. They were kept single housed under a 12-h light/12-h dark cycle. Food and water were given ad libitum. The EEG and EMG signals were sampled at 512 Hz, amplified and finally digitally filtered for storing at a frequency of 128 Hz. EEG and EMG channels were calibrated before each recording. The determination of the vigilance states (NREM, REM sleep and Wake) was carried out by off-line visual inspection of the EEG and EMG signals. Artifacts were left out from the EEG spectral analysis. Spectral analysis was done for each 4 s epoch in the frequency range 0.5–40 Hz using a resolution of 0.5 Hz.

We used the same analytical methods like Gradwohl et al. [27] and therefore in the following paragraphs of the “Methods” section (except “Data analysis and statistics” section) they are described shortly.

### Construction of a 2D state space

A 2D state space was determined by dividing 2 spectral amplitude ratios; ratio 1 of 0.5–20/0.5–40 Hz (plotted on the ordinate) and ratio 2 of 0.5–4/0.5–9 Hz (plotted on the abscissa) calculated at each sec. A sliding window Fourier transformation was used for each raw EEG signal (20 s wide Hanning window) of a 2 s window with an overlapping of 1 s step size. Distinct factors (e.g. type of animal and its age) influence these ratios and therefore to achieve the best separation of the vigilance states, we determined them heuristically by a careful examination of parameters for the numerator and denominator. The ratios were between 0 and 1, because the frequency range of the numerator was included in the denominator.

### State space densities

**Fig. 1:**
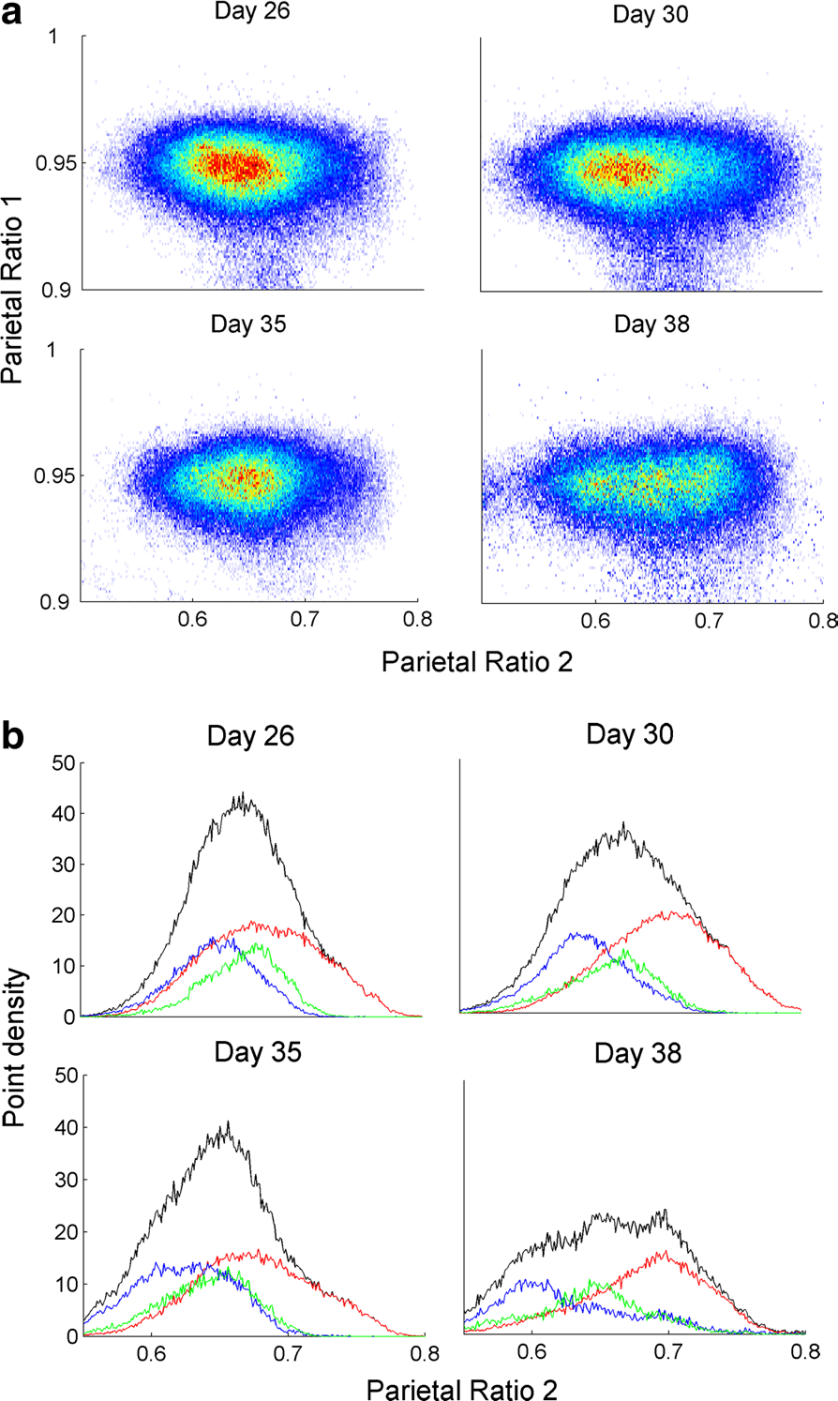
**a** point densities of 2D state space plots of an “average” animal with distinct clusters at the parietal lobe across 4 selected days. Each plot shows 6 h of EEG activity, and each point represents 1 s of EEG activity after application of a 20 s wide Hanning window. *Warm colors* indicate regions where the average density is high and *cool colors* indicate low average density. The *numbers in the color bar* are arbitrary. **b** “Average” state space densities at the 4 selected days, projected into ratio 2. Each of the vigilance state space point densities (not shown) was projected separately into ratio 2. *Blue* wake, *green* REM, *Red* NREM and *Black* summation of all vigilance sleep states. Note that the peak of the Wake vigilance state is located at lower ratio 2 than the peak of NREM

We produced the state space densities of the “average” animal (Fig. 1a; Additional file 1: Fig. S1a) by summing and averaging the density graph (not shown) of each animal using a grid of uniformly spaced boxes. High densities were indicated by warm colors and low densities by cold colors. Point densities were projected into ratio 2 (Fig 1b; Additional file 1: Fig. S1b). Based on the projection of each vigilance-state separately, we concluded that the peak of the Wake vigilance state is located at a lower ratio 2 than the peak of NREM sleep. In the 2D state space we organized the different vigilance states in clusters by using SST.

### State space movement velocities and trajectory/micro-arousal

The state space movement velocity for each rat was calculated upon the state space as the distance between two consecutive data points at a time window of 1 s. We organized the velocities to a grid which contained the mean value of all velocities originating at that point (not shown).

The trajectories were identified by following the movement of consecutive sequences of points between distinct clusters of the vigilance states in the state space. Each cluster contained all values up to 5% of the maximum value of this state. Trajectories were valid when the time duration of the trajectory between two clusters was 30 s, spending at least half of the preceding 15 secs in the source cluster and half of the subsequent 15 s in the destination cluster.

In addition, we explored changes in micro-arousal due to maturation during a 12 h window after light onset. A micro-arousal event was defined as a short trajectory lasting ≥3 up to 15 s from NREM or REM states to wake state.

### Data analysis and statistics

Sleep/wake dynamics and statistical analyses were performed using MATLAB (R-2013a, The MathWorks, Inc., Natick, MA, USA). Significance was analyzed using Student’s t tests for one level comparison or one factor ANOVA for comparison of more than 1 level. Two-way analysis of variance (ANOVA) with factors “region” (frontal/parietal) and factor “day” (P26, P30, P35, P38) for repeated measures was used to determine significant interactions between regions and days for velocity or trajectories/micro-arousals. Partial correlations, which rely on full correlation of the residuals, were calculated according to Pearson correlations, after excluding the effect of the independent factor “day”. To correct for multiple comparison new p-values were calculated according to the false discovery rate (FDR) in the case of statistical evaluation of different parameters on the same dataset [33]. The standard level for null hypotheses rejection remained 0.05. FDR correction was performed in Table 1 in the first part (excluding correlation), for 3 parameters (velocity, trajectory, micro-arousal) and in Table 2 for all 6 possible trajectories (Wake → NREM, NREM → Wake etc.). The number of rats (sample size) is n = 12 for the non-SD rats and n = 10 for SD rats.

**Table 1:** 2-way ANOVA of regional and age-related dependencies of EEG parameters in early and late sleep

	Comparison			
	Region		Day	
	First 6 h	Second 6 h	First 6 h	Second 6 h
Velocity	p < 0.00001, inter-p = 0.0498	p = 0.0002, inter-p = 1	p = 0.0061	p = 0.34716
Trajectory	p = 0.0011, inter-p = 1	p = 1, inter-p = 1	p = 1	p = 0.0087
Micro-arousals	p = 0.0015, inter-p = 1	p = 1, inter-p = 1	p = 0.0061	p = 0.0351

	Correlation			
	First 6 h		Second 6 h	
	Parietal	Frontal	Parietal	Frontal
Velocity-SWA	p = 0.0267, r = −0.4825	p = 0.3492, r = −0.2151	p = 0.7343, r = 0.0714	p = 0.945, r = 0.0149
Trajectory-SWA	p = 0.0608, r = 0.4900	p = 0.9361, r = 0.0236	p = 0.4104, r = −0.1686	p = 0.8347, r = 0.0430
Micro-arousal-SWA	p = 0.0029, r = −0.6752	p = 0.0234, r = −0.5042	p = 0.6479, r = 0.0940	p = 0.0475, r = 0.4000
Velocity-arousal	p = 0.0479, r = 0.3771	p = 0.0006, r = 0.6012	p = 0.0044, r = 0.5306	p = 0.0253, r = 0.4296

The new level for null hypotheses rejection was applied according to the false discovery rate (FDR). Interaction (Inter) means interaction between two curves according to 2-way ANOVA

**Table 2:** For all vigilance-states, all moving combinations of trajectories were calculated

Parietal				Frontal			
Direction	Mean	Stderr	%Total	Mean	Stderr	%Total	*T* test (parietal vs frontal)
*Trajectories*							
Total	107.00	1.65	100.00	39.78	2.04	100.00	
Wake → NREM	51.44	4.69	48.08	28.11	5.11	70.67	0.004
NREM → Wake	20.22	2.54	18.90	10.22	2.50	25.70	0.013
NREM → REM	18.00	1.79	16.82	1.00	0.52	2.51	0.000
REM → Wake	15.44	2.12	14.43	0.33	0.24	0.84	0.000
REM → NREM	1.56	0.38	1.45	0.11	0.11	0.28	0.002
Wake → REM	0.33	0.17	0.31	0.00	0.00	0.00	0.063
*Micro-arousal*							
Total	128.00		100	80.31		100	
NREM → Wake	102.85	6.00	80.35	76	8.36	94.63	0.013
REM → Wake	25.15	1.86	19.65	4.31	1.94	5.37	0.000

All trajectories are presented for the frontal and parietal lobes. In the parietal lobe, the total number of trajectories is 69% greater than in the frontal lobe. In both lobes, the most common trajectories were between Wake and NREM and vice versa. Other trajectories almost disappeared in the frontal lobe, in contrast to the parietal lobe. The total number of micro-arousals is 60% greater in the parietal lobe than in the frontal lobe. Most of the micro-arousal events in the parietal and frontal lobes occurred during NREM

## Results

### Vigilance states during maturation

**Fig. 2:**
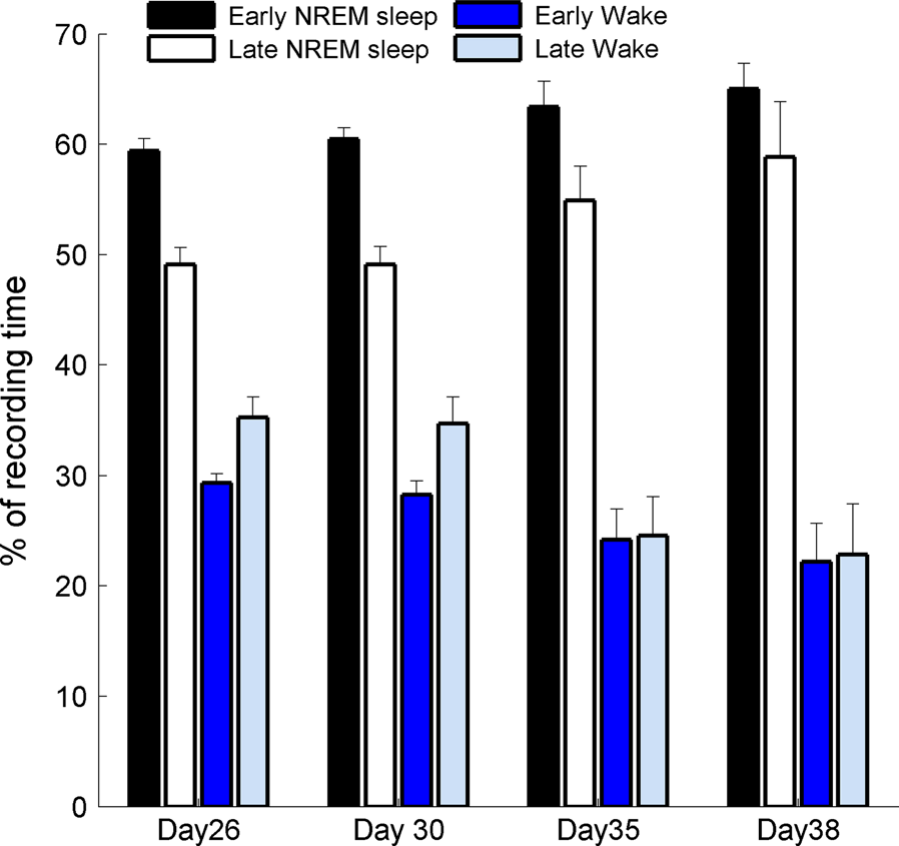
Maturational changes in NREM sleep and wake in rats. Values of NREM sleep and Wake are presented as a percentage of recording time. NREM sleep increases statistically (2-way ANOVA) versus maturation (p = 0.0094) and is larger in early sleep than in late sleep (p = 0.0011). Wakefulness decreases as a function of maturation (p = 0.0021)

We present here data of the first 12 h (as opposed to 3 h as in [11]) after light onset for vigilance-states during the day at four representative ages. These days are selected according to traditional methods for evaluating age-dependent changes in Wake, NREM, and REM sleep states. The amount of NREM sleep and Wake was examined for the first 6 h after light onset (early sleep), when sleep pressure is most prominent, and during the following 6 h (late sleep) after a decrease in sleep pressure. As a function of maturation, NREM sleep duration (Fig. 2) increased during early and late sleep, while the amount of Wake decreased. Moreover, the percent of NREM sleep during the first 6 h compared to the second 6 h was found to be higher on all selected days.

In the following paragraphs, state dynamics parameters were analyzed systematically to estimate age-dependent modifications of stability between early and late sleep. Furthermore, the age related state dynamics alterations were compared between the parietal and frontal lobe.

We extracted state dynamic parameters across early sleep and late sleep in the frontal and parietal lobes, which revealed noticeable changes of SWA in maturation in humans [7, 34] and rodents [11]. Here first some considerations related to the early and late sleep time window: During early sleep, sleep pressure is maximal, represented by high SWA. This maximal expression of SWA may partially mask age-dependent changes in SWA. However, in late sleep, when sleep pressure and SWA are decreased, changes due to maturation may be better expressed.

### Age related changes in dynamic state parameters and correlations

In the following paragraph we systematically analyzed the changes of state dynamic parameters versus maturation. More specifically, we explored two dependent parameters, recording electrode location (frontal or parietal) and time of sleep (early or late sleep) as a function of the independent parameter age. Therefore, the results of each dependent parameter as a function of age are presented separately:

#### Early and late sleep

**Fig. 3:**
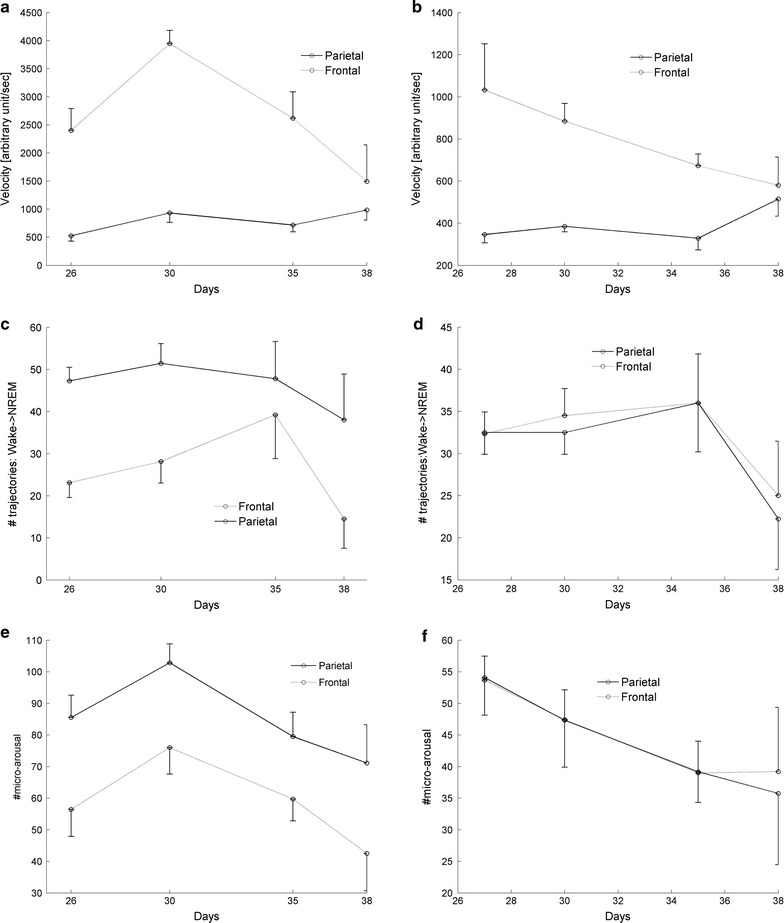
Dynamic EEG parameters in the frontal and parietal lobes in early sleep. The velocity in the frontal lobe was greater than in the parietal lobe in early (**a** p < 0.00001) and late sleep (**b** p = 0.0002). Moreover, in early sleep, velocity increased in the frontal lobe until day 30 and decreased over the following days. The same trend exists in late sleep, except at day 38. According to 2-way ANOVA, maturation dependency is significant (p = 0.0061). Regional differences in trajectories from Wake to NREM exist in early sleep (**b** p = 0.0011), while trajectory relates to age only in late sleep (**c** p = 0.0087). Micro-arousals from NREM to wake vigilance states show age-dependent alteration during early (**e** p = 0.0061) and late sleep (**f** p = 0.0351). During early sleep, the number of micro-arousals in the parietal lobe is significantly greater than in the frontal lobe (p = 0.0015)

In early sleep (at the frontal lobe), velocity as a function of maturation follows an inverted U-shaped curve, reaching maximal value at P30 before decreasing over the following days (Fig. 3a). In late sleep, no maturation dependent modifications of velocity were observed (Fig. 3b).

The number of trajectories versus maturation did not change in early sleep (Fig. 3c). In late sleep, after a decrease of sleep pressure, the number of trajectories changed significantly as a function of maturation (Fig. 3d), again showing an inverted U-shaped curve with maximal amplitudes at P30 and P35 in the parietal and frontal lobes, respectively.

In contrast to the velocity and trajectories, micro-arousals (from NREM to Wake) at early (Fig. 3e) and late sleep (Fig. 3f) showed a similar relationship to maturation. In early and late sleep, micro-arousals show age-dependent alterations following an inverted U-shaped time course. We choose micro-arousals from NREM to Wake as the representative parameters because the number of micro-arousals from REM to Wake are relatively small (see Table 2).

#### Frontal and parietal lobes

Differences between lobes remained stable across all ages. Velocity in the frontal lobe was faster relative to the parietal lobe (Fig. 3a, b). However, at both regions, the intra-regional velocities were uniform at all (ratio 1, ratio 2) coordinates at days 26, 30, 35 and 38 (not shown). In the frontal lobe the number of trajectories from Wake to NREM sleep was smaller than in the parietal lobe in early sleep (Fig. 3c). At the frontal and parietal lobe, the number of trajectories was similar in late sleep (Fig. 3d).

In accordance to the findings for trajectories, the number of micro-arousals at the frontal lobe was significantly smaller than in the parietal lobe (Fig. 3e) in early sleep. At both lobes, the number of micro-arousals in late sleep was similar (Fig. 3f).

**Fig. 4:**
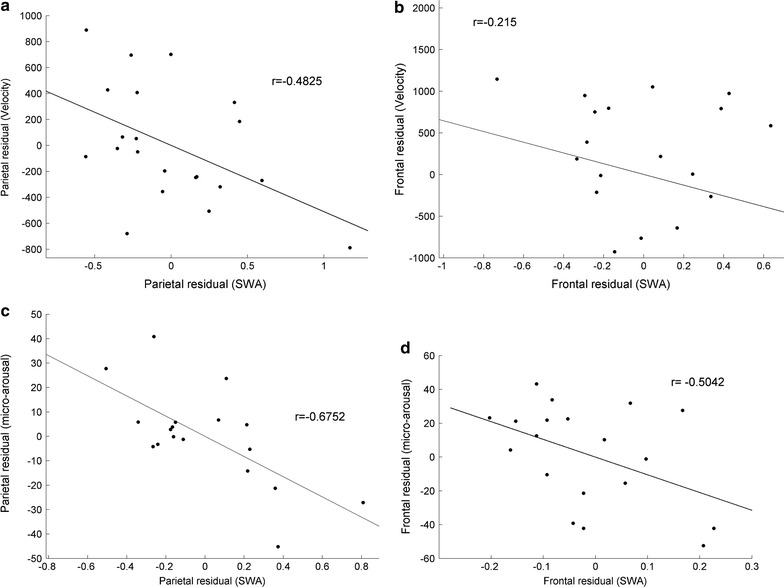
Correlations between residuals after age-excluded partial correlations between dynamic EEG parameters of SWA in early sleep. **a** In the parietal lobe, velocities are tend to be negatively correlated to SWA (p = 0.0559), while no significant correlations exist in the frontal lobe (**b**). **c** Negative correlation exists between micro-arousals and SWA in the parietal lobe (p = 0.0087), and frontal lobe (p = 0.0498) (**d**)

**Fig. 5:**
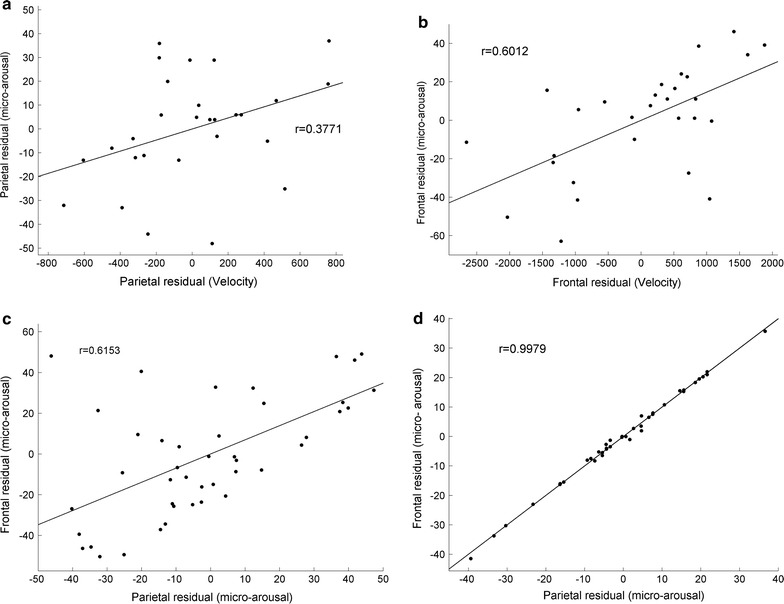
Correlations of residuals between dynamic EEG parameters after age exclusion in early sleep. **a** Positive partial correlations between micro-arousals and movement velocities tend to be significant in the parietal lobe (p = 0.0628) and are significant in the frontal lobe (p = 0.0024) (**b**). **c** Micro-arousals are significantly correlated between the parietal and frontal lobes (p < 0.0001) in early sleep and this correlation increases in late sleep (**d** r = 0.9979, p < 0.00001)

We investigated the relationship, between SWA and the state dynamic parameters and in-between the state dynamic parameters themselves, by partial correlation since the factor “age” may mask a potential correlation. Velocity showed a tendency for a relationship with SWA in the parietal lobe (Fig. 4a, p = 0.0267, r = −0.4825 in early sleep) while no correlation existed in the frontal lobe (Fig. 4b). No correlations occurred between the trajectories and any other parameters. Negative correlations existed between micro-arousals and SWA in the parietal lobe in early sleep (Fig. 4c, p = 0.0029, r = −0.6752) but not in the frontal lobe (Fig. 4d). Finally, during early sleep, positive correlations were observed between micro-arousals and velocities in the parietal lobe (only trend, Fig. 5a, p = 0.0479, r = 0.3771) and in the frontal lobe (Fig. 5b, p = 0.0006, r = 0.6012). In late sleep, micro-arousals in the parietal lobe correlated positively with velocity (p = 0.0044, r = 0.5306). These statistics are summarized in Table 1. Micro-arousals in both lobes correlate in early sleep (Fig. 5c, p < 0.00001, r = 0.6153) and this correlation increases considerably in late sleep (p < 0.00001, r = 0.9979).

### Trajectories and micro-arousals between vigilance-states at P30

To analyze patterns of 2D state space dynamics as rats move between vigilance states, we identified trajectories in 2D state space by tracking consecutive sequences of points at P30, the day with the largest number of micro-arousal in the early sleep (Fig. 3e). Using a contour algorithm (see "Methods" section ), we delineated cluster core boundaries for each possible vigilance-state (Table 2). For all vigilance-states, all moving combinations of trajectories were calculated at day 30 with the most prominent SWA. All trajectories are presented in the frontal and parietal lobes. In the parietal lobe, the total number of trajectories was 69% greater than in the frontal lobe. In both lobes, the most common trajectories were between Wake and NREM sleep: 48.00 and 70.67% and vice versa 18.90 and 25.70% in the parietal and frontal lobes, respectively. Other trajectories almost disappeared in the frontal lobe, in contrast to the parietal lobe (see Table 2).

The total number of micro-arousals in the parietal lobe was 60% greater than in the frontal lobe. Most of the micro-arousal events in the parietal and frontal lobes, 80.35 and 94.63%, respectively, occurred during NREM sleep.

### Are movement velocities, trajectories and micro-arousals related to the homeostatic regulation?

**Fig. 6:**
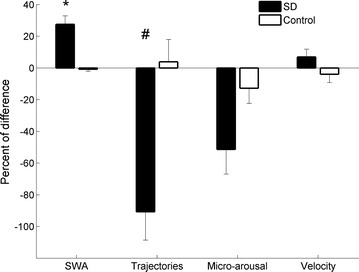
In the frontal lobe, we compared the EEG parameters of sleep deprivation (SD) rats relative to the previous day without SD and calculated the percent difference. We performed the same comparison between the same days and hours for control rats without SD. SD enhances SWA by 27.5% (p = 0.0014) relative to the previous day while trajectories decreased by 90.8% (p = 0.0027). The decrease in micro-arousals by SD is not significant (p = 0.0792) and velocity does not change due to SD (p = 1). No change occurred in the control rats

State dynamic parameters followed an inverted U-shaped curve during maturation like SWA (as shown in the previous section). Because SWA is controlled by the homeostatic regulation of sleep, we asked if the changes of the state dynamic parameters also depend on this regulatory system. To explore this question, EEG parameters were measured after a short period of SD. We based our analysis on the theory that if EEG parameters were modified due to SD, then they rely on the homeostatic component. If, however, the EEG parameters were not modified by SD, then they are modulated by another factor. To explore this hypothesis, rats aged P32–P36 were sleep deprived for 4 h. Then EEG recordings were analyzed for the following 3 h while the effect of SD was maximal. We compared the EEG parameters in the frontal lobe of SD rats relative to the corresponding time interval during the previous baseline day and calculated the percent difference (Fig. 6). We performed the same comparison between the same days and hours for control rats without SD. SD enhanced SWA by 27.5% relative to the previous day while the trajectories decreased by 90.8%. The decrease in micro-arousals by SD was not significant and the velocity did not change due to SD. No changes occurred in rats during control days.

## Discussion and conclusions

In this study, we present evidence, using an unbiased SST method, that maturation in rats produces noticeable temporal alterations in ‘dynamic’ EEG parameters in sleep/wake activity, which cannot be explored by conventional sleep analysis methods. We showed that trajectories and micro-arousals, changed age-dependently following an inverted U-shape curve in the frontal and parietal lobes The time course of movement velocities are only in the frontal lobe U-shaped. At all ages velocity at the frontal lobe had larger values in comparison to the parietal lobe, while trajectories and micro-arousals in the parietal lobe were larger than in the frontal lobe. It is important to note that velocity and micro-arousal are negatively correlated to SWA, and, therefore, velocity and micro-arousal are positively correlated. Our analysis shows that it is probable that, as known for SWA, trajectories are regulated by the homeostatic component of sleep. Movement velocities and micro-arousals, on the other hand, seem rather to depend on other factors, for example circadian rhythms. Despite the negative correlation between SWA and velocity, as well as between SWA and micro-arousal, also these parameters showed an inverted U-curve during maturation when controlling for “age” when using partial correlations.

### Age-related U-curve of EEG parameters

In humans, SWA follows an inverted U-shape trajectory, i.e. increases in the first years of life, reaches a maximal value around puberty and declines throughout adolescence [11, 13, 35–37]. Olini et al. [11] revealed that SWA increases after birth until peaking at P30, then decreases as animals age. As we show here state dynamics parameters of velocity, trajectories and micro-arousals are related to maturation in a similarly inverted U-shape, with a peak at P30 or P35. In a previous study [27],evidence was presented that no velocity differences exists in rats at P34 compared to P71. Thus maturational changes in these parameters may be finalized around P35. This interpretation is supported by an electron microscopy study showing that synaptic density in rats was reaching stable levels after about 1 month of age [38]. In humans, the time course of sleep SWA seems to be related to cortical gray matter maturation [39]. Other studies [40–43] proposed a relationship between SWA and cortical gray matter, without an age related dependency of these parameters. On a functional level, such a relationship between SWA and gray matter might be explained by changes in network synchronization due to age dependent changes in network connectivity. The velocity, trajectories and micro-arousals seem to be also influenced by this age dependent changes in connectivity.

### Regional differences in EEG parameter efficiency and maturation

Regional differences in sleep dependent parameters were sustained over all ages of maturation. Several studies reported that SWA, as a consequence of neuronal population activation, is not equal over the cortex but is region dependent [44–46]. A possible explanation may be that the neuronal connectivity in the frontal lobe shows a denser presence of neurons than the parietal lobe [47], and thus, modifying neuronal synchronization and synaptic strength in NREM sleep [48]. Additionally, it is known that in rats, the frontal lobe reflects heavier neuronal use/plasticity due to distinct motor [49] and learning stimulations. Local enhancement or reduction of SWA seems to be modulated by local, use-dependent processes of plasticity in the preceding wake state [50], as reflected by local potentiation after learning new tasks [51, 52] or by synaptic depression after arm immobilization in humans [53].

Because micro-arousals in the parietal and frontal lobes are significantly positive correlated, the system of controlling micro-arousals in these regions seem to be equal. However, the number of parietal micro-arousals is greater than in the frontal lobe. We assume that increased sleep pressure (as reflected by increased SWA) tends to decrease the micro-arousal system and, in accordance with the negative correlation between micro-arousals and SWA, as shown here and elsewhere [54], micro-arousals are smaller in the frontal lobe in comparison to the parietal lobe. We report here that movement velocities in the frontal lobe are faster than in the parietal lobe, despite high SWA in the frontal lobe. A potential mechanism might be that fast velocities during high SWA can also contribute to stability, similar to micro-arousals and trajectories, and can enable the brain to sustain an effective NREM sleep vigilance state for as long as possible. In other words, due to high velocities, the EEG returns to NREM sleep quickly after diverting to other vigilance states. Since recovery processes take place during NREM sleep, staying as long as required and continuously in NREM sleep might be essential.

### Slow wave activity mask

In this study, we analyzed EEG modification during maturation and attempted to separate the homeostatic component from other modulating factors. However, high SWA may mask the maturational changes due to ceiling effects. Therefore, our idea for analyzing EEG-related parameters during early and late sleep was based on the assumptions that age-related changes could be underestimated during early sleep when SWA or sleep pressure is maximal and that late sleep would reveal these alterations as sleep pressure declines considerably. Indeed, all homeostatic EEG parameters (SWA and trajectories) changed in late sleep as a function of maturation. As a marker of the homeostatic regulation of sleep, SWA increases after SD, largely independent of circadian phase [55]. Thus if one of the parameters changed as a consequence of SD, we concluded that it was primarily related to the homeostatic component; otherwise the parameter was rather related to other factors, like circadian rhythms [55]. Based on these assumptions trajectories are considered to be controlled by the homeostatic component of sleep and movement velocities/micro-arousals seem to be mainly affected by other factors.

### Study limitations

SST is especially useful for analyzing the dynamics of sleep/wake instability, but has some limitations, as previously discussed [27–29]. In our study, large variability was observed in the SST-based EEG parameters. This finding is in accordance with previous reports [27–29] and further studies are required to explore this issue. Using frequency ratios of parameters could yield hypersensitivity, and small changes in the denominator over time may lead to huge fluctuations in the corresponding ratio. Another limitation can be that the density graphs yield distinct boundaries at different hours. Therefore, it should be recognized that comparison of trajectories and micro-arousals between different hours could be problematic because values are calculated according to these boundaries. Additionally, separation between the Wake and REM sleep clusters was less well-defined using surface EEG signals [31]. Another limitation is that the effect of maturation is not consistently showing significant effects in the frontal or parietal lobes during early or late sleep. As discussed in the previous paragraph, a masking effect could explain this phenomenon.

### Possible consequences of this study

In this study, we explored distinct EEG parameters and demonstrated that, as found for SWA, also SST parameters, like trajectories and micro arousals, show age dependent changes which are modulated by region. These dynamic parameters presented here might therefore provide additional valuable insights into the close relationship between sleep and maturational process. This relationship is of particular relevance because increasing evidence supports an active role of sleep in plasticity processes [56–61] and adolescence is recognized as a time period during which the incidence of psychiatric disorders including mood disorders, anxiety and schizophrenia is dramatically increasing [62]. In fact 50% of all life time mental disorders develop before the age of 14 years [63]. At the same time the incidence of sleep disorders increases [64]. Thus, if sleep plays an active role in maturational processes we should engage the whole repertoire of analysis tools to discover aberrant maturation of sleep. Early diagnosis of mental disorders can be promoted by exploring the sleep stability by SST, in addition to the traditional method.

## Additional file

**Additional file 1: Fig. S1.** **a** Point densities of 2D state space plots of an “average” animal with distinct clusters at the frontal lobe across 4 selected days. Each plot shows 6 h of EEG activity, and each point represents 1 s of EEG activity. Warm colors indicate regions where the average density is high and cool colors indicate low average density. The numbers in the color bar are arbitrary. **b** “Average” state space densities on 4 selected days, projected into ratio 2. Each of the vigilance state space point densities (not shown) was projected separately into ratio 2. Blue—Wake; Green—REM; Red – NREM and Black—summation of all vigilance sleep states

## Abbreviations

EEG: electroencephalogram
SWA: slow-wave activity
SST: state space technique
NREM: non-rapid eye movement
REM: rapid eye movement
SCN: suprachiasmatic nuclei
GluR1: glutamate receptor subunit R1
AMPA: α-amino-3-hydroxy-5-methyl-4-isoxazolepropionic acid
ASDA: American Sleep Disorders Association
2D: 2-dimensional
EMG: electromyogram
SD: sleep deprivation
ANOVA: two-way analysis of variance
FDR: false discovery rate
Inter: interaction
