# Supplementary figures and images for: Sleep/wake movement velocities, trajectories and micro-arousals during maturation in rats
Source: BMC Neurosci. 2017 Feb 7;18:24. doi: 10.1186/s12868-017-0343-6 (PMC5297220; doi:10.1186/s12868-017-0343-6)

Fig. S1a


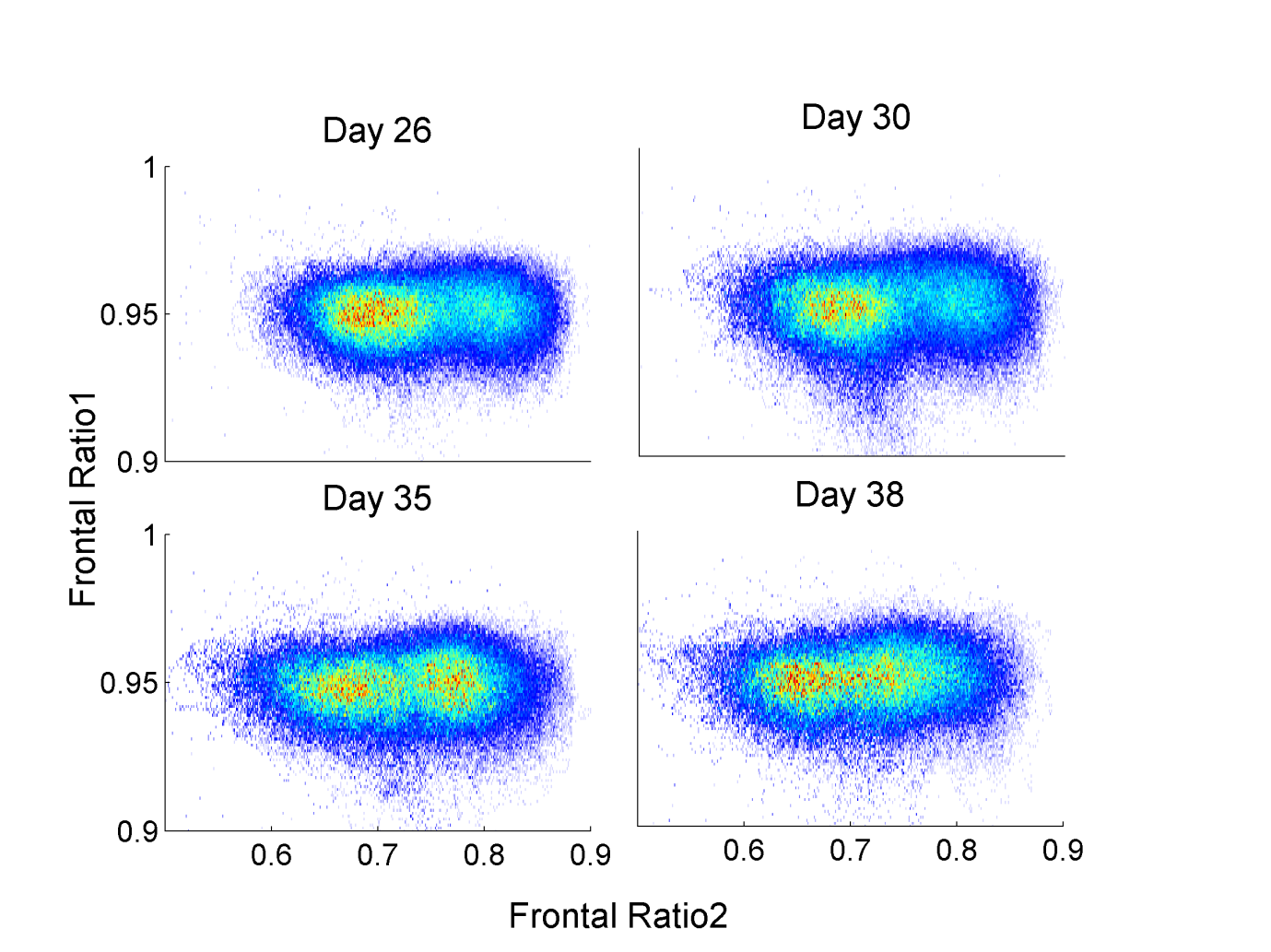


Fig. S1b


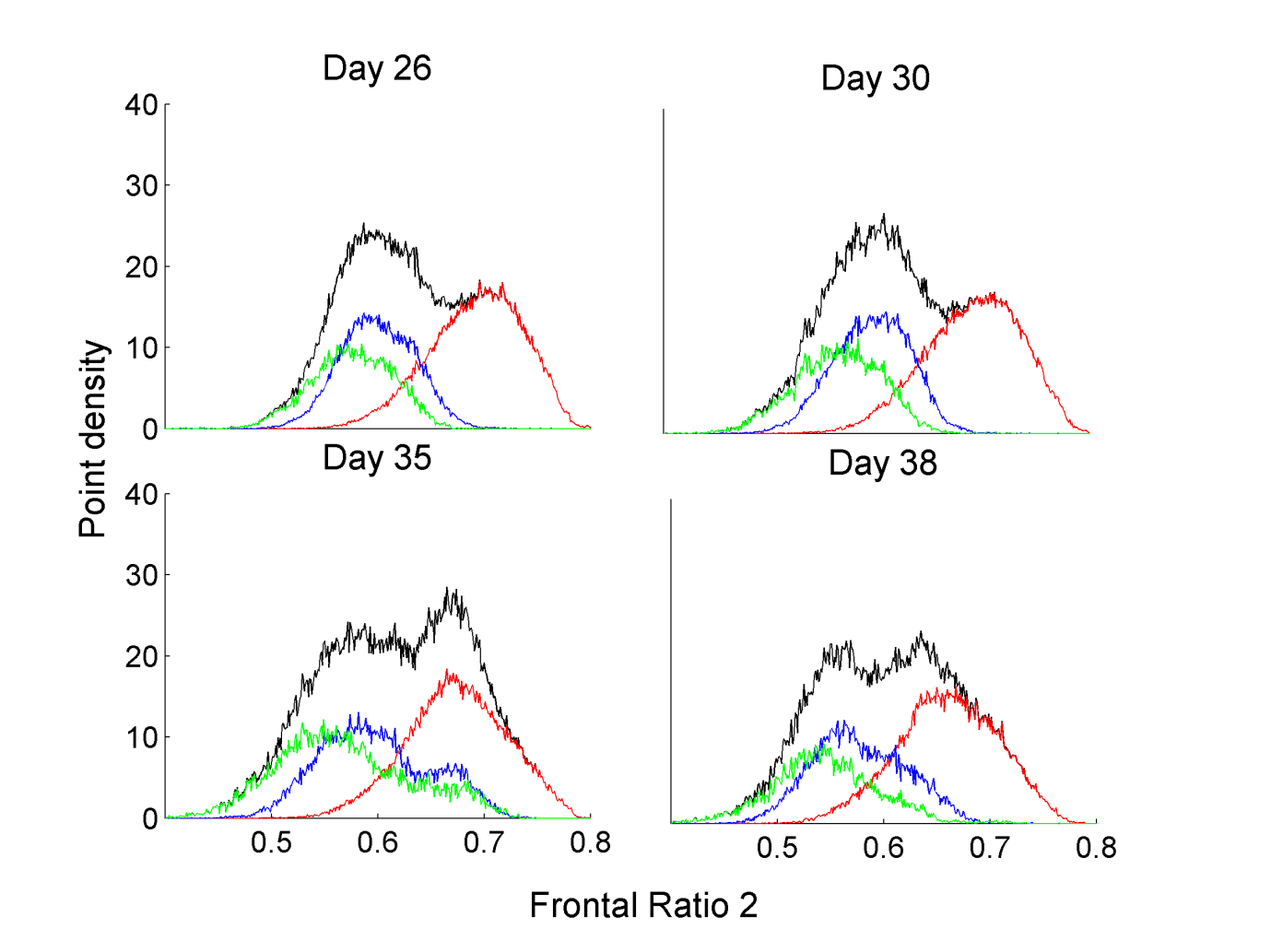

Supplement: Supplementary file 1 — Additional file 1: Fig. S1. a Point densities of 2D state space plots of an “average” animal with distinct clusters at the frontal lobe across 4 selected days. Each plot shows 6 h of EEG activity, and each point represents 1 s of EEG activity. Warm colors indicate regions where the average density is high and cool colors indicate low average density. The numbers in the color bar are arbitrary. b “Average” state space densities on 4 selected days, projected into ratio 2. Each of the vigilance state space point densities (not shown) was projected separately into ratio 2. Blue—Wake; Green—REM; Red – NREM and Black—summation of all vigilance sleep states [file 12868_2017_343_MOESM1_ESM.docx]
